# Nutraceuticals from Algae: Current View and Prospects from a Research Perspective

**DOI:** 10.3390/md20110671

**Published:** 2022-10-26

**Authors:** Brijesh K. Tiwari, Marco Garcia-Vaquero

**Affiliations:** 1 Teagasc Food Research Centre, Ashtown, Dublin 15, D15 DY05 Dublin, Ireland; 2 Section of Food and Nutrition, School of Agriculture and Food Science, University College Dublin, Belfield, Dublin 4, D04 V1W8 Dublin, Ireland

## Introduction

In recent years, algae, both microalgae and macroalgae, have attracted the attention of the scientific community as a source of multiple active molecules or bioactives, including polysaccharides, fatty acids, proteins and peptides, polyphenols, diterpenes, steroids, and alkaloids. Compared with other natural sources, marine organisms have taken the lead in the discovery of new drugs. Oceans cover more than 70% of the Earth’s surface and represent a challenging environment for the growth of marine organisms, with extreme fluctuations in water level, solar radiation, and temperature; an environment in which algae are able to thrive by producing unique metabolites, different in composition from those of terrestrial plants, which play a major role in the protection of biomass [1]. The unique chemical features of algal compounds and their reported health benefits have contributed to expanding the interest in these molecules far beyond their pharmaceutical and cosmeceutical applications, with the food industry gaining interest in the incorporation of these molecules as functional foods or nutraceuticals [2]. Innovative technologies and processes are currently being explored to improve the use of algae as a source of nutraceuticals. The future use of algal biomass for food and nutraceutical purposes needs a dynamic research environment to understand the knowledge gaps and develop new strategies for the optimum exploitation of these resources, including the improved production/cultivation and understanding of the biomass, the sustainable and green extraction of high-value compounds, and finally, exploring the biological properties of these compounds using in vitro, ex vivo, and in vivo models. Further multidisciplinary research is needed to establish new or improve current methods for algae production, including the application of stressors or manipulations of the biomass targeting the overproduction of high-value compounds; as well as analyzing the changes in composition of the wild biomass to establish a relationship between the stressors and compositional changes in algae that can guide their future culture and industrial exploitation. Moreover, the use of innovative and emerging technologies, including ultrasound, microwaves, electric fields, and high-pressure and supercritical fluids, have also shown promising results for the development of efficient and green extraction, isolation, purification, and preservation processes of algal compounds for their future use as nutraceuticals. Finally, the health benefits of these compounds will also have to be demonstrated using in vitro, ex vivo, and/or in vivo model systems to enable the future commercialization of marine compounds as nutraceuticals and to establish health claims. This Special Issue contains nine articles, including five research articles and four reviews, covering multiple innovative aspects of the exploitation of algae for the development of nutraceuticals. Here, we provide a brief overview of what the reader will find in this Special Issue.

Wang et al. [3] reviewed and proposed improvements to adaptive laboratory evolution (ALE), an innovative method to explore strain improvements for microalgal production in developing new biological and phenotypic functions and improving the performance of strains in microalgal biotechnology. The authors identified several challenges and proposed solutions for improvements of ALE, including the generation of a high-quality mutant library to identify the genetic diversity of ALE, the need for multi-omics to promote the efficient data mining and implementation of ALE experiments, and the need to incorporate novel cultivation strategies, such as red LED light and phytohormones, to accelerate the ALE process, amongst other strategies that will need upgraded and developed software for more effective data interpretation [3].

Bolaños-Martínez et al. [4] provided an update on microalgal biotechnology strategies for pharmaceutical applications, including techniques for the generation of recombinant proteins and genetic engineering processes, including viral-based vector constructions. The use of viral vectors is relatively new in algae, and they are gaining momentum for the production of biopharmaceuticals because they have higher yields and shorter production times compared with chloroplast and nuclear-stable transformation methods. The authors also emphasized that in February 2022, one company produced the first COVID-19 vaccine in plants (COVIFENZ^®^) that was approved by the Health Agency in Canada, fact that can be a stepping-stone for the green production of these products for human use [4].

Čagalj et al. [5] generated extracts from *Cystoseira compressa* collected in the Central Adriatic Sea using microwave-assisted extraction technology from algae collected during the seasonal growth period (May–September). The authors analyzed the total phenolic content, total tannin content, antioxidant activities (measured as ferric reducing antioxidant power (FRAP), 2,2-diphenyl-1-picrylhydrazyl radical scavenging activity (DPPH) and oxygen radical absorbance capacity (ORAC)), and the antimicrobial effect of these compounds against *Listeria monocytogenes*, *Staphylococcus aureus*, and *Salmonella enteritidis*. The authors reported the highest antibacterial activity in extracts generated in June, July, and August, and associated those results with compounds produced when the sea temperature was at its highest. Moreover, the authors emphasized the usefulness of the *C. compressa* biomass as a source of nutraceuticals [5].

Meinita et al. [6] reviewed the biological activities, as well as the safety and toxicity levels of fucosterol from marine algae, relevant for the use of these compounds in the nutraceutical and pharmaceutical industries. The literature search focused on the period 2002–2020, and following the Preferred Reporting Items for Systematic Reviews and Meta-Analyses method, the authors identified 43 studies that could help to fill certain research gaps. Overall, the review concluded that fucosterol exhibited low toxicity in animal cell lines, human cell lines, and animals. However, the authors emphasized the need for further safety and toxicity reports of this compound under clinical settings [6].

O’Connor et al. [7] reviewed the current and emerging processes for the generation of algal bioactive peptides, including pre-treatments for the extraction of protein from algae and methods for the generation of hydrolysates and purification of these compounds. The authors outlined the main biological properties attributed to determining bioactive peptide sequences isolated from algae, including anti-hypertensive, antioxidant, and anti-proliferative/cytotoxic effects assayed in vitro and/or in vivo, and emphasized the use of in silico tools, such as quantitative structural activity relationships (QSARs) and molecular docking, as powerful tools to accelerate the discovery of these promising compounds [7].

Ahmed et al. [8] reviewed the potential of marine bioactive peptides for human health due to their unique chemical structures, physicochemical, and biological activities. The authors focused on marine microorganisms, including microalgae, bacteria, and fungi, considered as good sources of amino acids and peptides, and emphasized the relevance of the marine biome and the opportunities it offers for the valorization of marine-biome-based bioactive peptides. The review also summarizes FDA-approved marine bioactive peptides, as well as the legislation, challenges, and future perspectives for the increased use of these compounds [8].

Garcia-Vaquero et al. [9] focused on experimentally exploring the application of innovative extraction technologies (ultrasound-assisted extraction (UAE), microwave-assisted extraction (MAE), ultrasound–microwave-assisted extraction (UMAE), hydrothermal-assisted extraction (HAE), and high-pressure-assisted extraction (HPAE)). The authors used fixed extraction conditions (solvent: 50% ethanol; extraction time: 10 min; algae/solvent ratio: 1/10) when using all the selected innovative technologies, and they explored the application of a time post-treatment (0 and 24 h) for the recovery of phytochemicals (total phenolic content, total phlorotannin content, total flavonoid content, total tannin content, and total sugar content) and associated antioxidant properties (DPPH and FRAP) from *Fucus vesiculosus* and *Pelvetia canaliculata*. Overall, UAE generated extracts with the highest phytochemical contents from both macroalgae, with the highest yields of compounds generated from *F. vesiculosus* which included the total phenolic content (445.0 ± 4.6 mg gallic acid equivalents/g), total phlorotannin content (362.9 ± 3.7 mg phloroglucinol equivalents/g), total flavonoid content (286.3 ± 7.8 mg quercetin equivalents/g), and total tannin content (189.1 ± 4.4 mg catechin equivalents/g). The DPPH antioxidant activities were at the highest levels in extracts generated by UAE and UMAE from both macroalgae, whereas no clear pattern was appreciated for FRAP. Moreover, the authors determined that after the application of these innovative technological treatments, additional storage post-extraction did not improve the yields of phytochemicals or antioxidant properties of the extracts [9].

Elhady et al. [10] isolated three fatty acids (docosanoic acid 4, hexadecenoic acid 5, and alpha hydroxy octadecanoic acid 6) and three ceramides (A (1), B (2), and C (3)) from the macroalga *Hypnea musciformis*. The authors analyzed the biological properties of these isolated compounds and determined that ceramides A (1) and B (2) had in vitro cytotoxic activity against human breast adenocarcinoma (MCF-7) cell lines. Furthermore, when assayed in vivo using a mouse model of Ehrlich ascites carcinoma (EAC), both compounds reduced the size of the tumors in inoculated mice: in the case of ceramide A (1) at a dose of 1 mg/kg; and in the case of B (2) at a dose of 2 mg/kg. Overall, the authors’ findings demonstrated the cytotoxic, apoptotic, and antiangiogenic effects of ceramides from *Hypnea musciformis* [10].

Wei et al. [11] researched the effects of polysaccharides extracted from the macroalgae *Sargassum fusiforme* by water extraction (SfW) and acid extraction (SfA) on the cecal and fecal microbiota of mice fed high-fat diets (HFDs) by 16S rRNA gene sequencing. The authors determined that 16 weeks of HFD administration dramatically impaired the homeostasis of both the cecal and fecal microbiota, without affecting the relative abundance of *Firmicutes*, *Clostridiales*, *Oscillospira*, and *Ruminococcaceae* in cecal microbiota and the Simpson’s index of fecal microbiota. Co-treatments with SfW and SfA exacerbated body weight gain and altered the abundance of genes encoding monosaccharide-transporting ATPase, α-galactosidase, β-fructofuranosidase, and β-glucosidase with the latter showing more significant potency. Overall, the authors concluded that SfW and SfA could regulate the cecal microbiota, pointing out the relevance of further studies on the influence of macroalgal polysaccharides as gut microbiota regulators [11].
